# miR-340-5p Alleviates Oxidative Stress Injury by Targeting MyD88 in Sepsis-Induced Cardiomyopathy

**DOI:** 10.1155/2022/2939279

**Published:** 2022-05-04

**Authors:** Cong Zhang, Lijin Zeng, Guoyi Cai, Yuanting Zhu, Yan Xiong, Hong Zhan, Zhen Yang

**Affiliations:** ^1^Division of Emergency Medicine, Department of Emergency Intensive Care Unit, The First Affiliated Hospital, Sun Yat-sen University, Guangzhou 510080, China; ^2^Department of Cardiology, The First Affiliated Hospital, Sun Yat-sen University, Guangzhou 510080, China; ^3^NHC Key Laboratory on Assisted Circulation (Sun Yat-sen University), Guangzhou 510080, China

## Abstract

**Background:**

Sepsis-induced cardiomyopathy (SIC) is a sort of severe disease in the intensive care unit. This research focuses on exploring the influence of miR-340-5p on SIC and its specific mechanism.

**Methods:**

Mice were administered with lipopolysaccharide (LPS) to construct a SIC animal model. Mice were intramyocardially injected with Adenoassociated Virus- (AAV-) 9 containing the miR-340-5p precursor to make the miR-340-5p overexpression in the myocardium. The expression level of myocardial miR-340-5p was evaluated by qRT-PCR. The cardiac function was measured by echocardiography, the myocardial morphology was observed by hematoxylin-eosin (HE) staining, and the oxidative stress level was detected by 4-hydroxynonenal (4-HNE) immunohistochemical staining and malondialdehyde (MDA) assay in mice. The cells were pretreated with miR-340-5p mimic, mimic-NC, miR-340-5p inhibitor, inhibitor-NC, MyD88 siRNA, or si-NC and then administered with LPS or PBS. The cell viability was measured with the CCK-8 assay. The level of intracellular oxidative stress was evaluated using reactive oxygen species (ROS), MDA, and glutathione (GSH) detection. The MyD88 level was assessed via Western blotting analysis. The interaction of miR-340-5p with the MyD88 mRNA was confirmed via dual-luciferase reporter assay and RNA pull-down assay.

**Results:**

The miR-340-5p overexpression partially alleviated the increase of the MyD88 level, impairment of cardiac function, and oxidative stress injury in the SIC animal model. In the SIC cell model, miR-340-5p mimic pretreatment partially relieved oxidative stress injury, while the miR-340-5p inhibitor had the opposite effect. Besides, the miR-340-5p mimic and inhibitor could reduce and further increase the MyD88 level in the SIC cell model, respectively. Dual-luciferase reporter and RNA pull-down experiments confirmed the interaction between the MyD88 mRNA and miR-340-5p. Finally, it was found that MyD88 siRNA pretreatment also partially alleviates the oxidative stress injury in the SIC cell model.

**Conclusion:**

In sum, our study demonstrated that miR-340-5p can improve myocardial oxidative stress injury by targeting MyD88 in SIC.

## 1. Introduction

Sepsis is a common fatal disease in the intensive care unit, which is characterized by severe organ dysfunction syndrome induced by the imbalance of host response to infection [1, 2]. The previous research has shown that the number of cases of sepsis reaches 48 million every year in the world, of which more than a quarter will eventually die [3]. Sepsis can cause injury to the heart, brain, liver, kidney, and other organs, among which cardiac dysfunction is a major cause of patient death [4, 5]. Compared with the patients without cardiac dysfunction (20%), the mortality of sepsis-induced cardiomyopathy patients (70%-90%) was significantly higher [6]. Sepsis-induced cardiomyopathy (SIC) is commonly manifested via left ventricular ejection fraction deficiency, ventricular enlargement, and reversible functional injury, and its pathogenesis has not been completely clear until now [1, 7]. Therefore, exploring the new molecular mechanisms to obtain new targets will contribute to the treatment of SIC in the future.

MicroRNAs are short noncoding RNAs widely existing in organisms, which are characterized by their good conservation among species [8]. miRNAs mainly function at the posttranscriptional level and affect various pathophysiological processes such as organogenesis, severe inflammation, and tumorigenesis [9, 10]. Many studies have shown that multiple miRNAs (miR-21-3p, miR-125b, and miR-146b, etc.) may affect the occurrence and development of SIC [11–13]. Besides, some other researchers have found that miR-340-5p has diverse effects on different cardiac diseases (myocardial ischemia-reperfusion, heart failure, and myocardial hypertrophy, etc.) [14–18]. Moreover, a bioinformatics analysis study suggested that miR-340-5p has an effect on sepsis-induced renal injury [19]. However, whether miR-340-5p functions in SIC is still unclear.

Myeloid differentiation primary response 88 (MyD88) is a key molecule in the Toll-like signaling pathway, which plays a vital role in the innate and adaptive immune response [20, 21]. AbdAllah et al. have found that a high circulatory MyD88 level is significantly correlated with the poor prognosis of neonatal sepsis [22]. Our previous transcriptome sequencing result analysis on a LPS-treated mouse heart suggested that the MyD88 gene is a key node gene of SIC [23]. In addition, through bioinformatics analysis, we predicted that miR-340-5p could interact with MyD88 3′UTR, and the site is conservative in different species. Therefore, we hypothesize that miR-340-5p can improve SIC by targeting MyD88.

In this study, we used lipopolysaccharide- (LPS-) treated mice or HL-1 cells to construct a sepsis-induced cardiomyopathy model, observed the miR-340-5p and MyD88 expression levels, detected the influence of miR-340-5p overexpression or knockdown and MyD88 siRNA on the SIC model, and explored its specific mechanism.

## 2. Materials and Methods

### 2.1. Animal Experiments

This research was approved and supervised by the Institutional Animal Care and Use Committee of Sun Yat-sen University. The 5–8-week-old C57BL/6J mice were purchased from GemPharmatech (Jiangsu, China). All mice were fed in an environment with sufficient food and water freely accessible, constant temperature, and appropriate humidity under a 12-hour light/dark cycle. The animals were divided into four groups: the control group, LPS group, miR-340-5p group, and miR-340-5p-combined LPS group. As previously described, mice were intraperitoneally injected with LPS (Sigma-Aldrich, 10 mg/kg) for 6 hours to establish the sepsis-induced cardiomyopathy model [23]. In the control group (OE-NC group), the mice were intramyocardial injected with 20 *μ*l (1 × 10^11^ vg/mice) of Adenoassociated Virus- (AAV-) 9 negative control (Shanghai GeneChem Co., Ltd., China) at day 0 and were intraperitoneally injected with phosphate-buffered saline (PBS) buffer for 6 hours at day 21. The LPS group (OE-NC+LPS group) mice were intramyocardial injected with 20 *μ*l (1 × 10^11^ vg/mice) AAV-9 negative control at day 0 and were intraperitoneally injected with 10 mg/kg LPS for 6 hours at day 21. The miR-340-5p group (OE-miR-340-5p group) mice were intramyocardial injected with 20 *μ*l (1 × 10^11^ vg/mice) AAV-9 pre-miR-340-5p (Shanghai GeneChem Co., Ltd., China) at day 0 and were intraperitoneally injected with PBS for 6 hours at day 21. In the miR-340-5p-combined LPS group (OE-miR-340-5p+LPS group), animals were intramyocardial injected with 20 *μ*l (1 × 10^11^ vg/mice) AAV-9 pre-miR-340-5p at day 0 and were intraperitoneally injected with 10 mg/kg LPS for 6 hours at day 21. Echocardiographic measurement was performed on the mice, or they were sacrificed to obtain the heart tissue.

### 2.2. Cell Culture and Transfection

According to the instructions, HL-1 cells (iCell Bioscience Inc., Shanghai, China) were plated in minimum essential medium (MEM) containing 10% fetal bovine serum (Gibco, NY, USA) and 1% penicillin-streptomycin (Gibco, NY, USA), and they were maintained in an incubator at 37°C and 5% CO_2_. Firstly, the HL-1 cells were placed into six-well plates. Then, the miR-340-5p mimic, mimic-NC, miR-340-5p inhibitor, inhibitor-NC, MyD88 siRNA, or si-NC (GenePharma, Shanghai, China) were transfected into the cells through a Lipofectamine 2000 kit (11668-027; Invitrogen, CA, USA) when the cell confluence reached 30%-50%. After transfection for 4 hours, the old medium was replaced with a fresh complete medium. 48 hours after transfection, the HL-1 cells were administered with LPS (10 *μ*g/ml, 6 hours) and the same amount of PBS to construct the sepsis-induced cardiomyopathy cell model and control group, respectively. The sequences of the mimic, inhibitor, siRNA, or control are shown in Table 1.

### 2.3. Echocardiography

As mentioned in previous studies [24, 25], echocardiography was performed using the Vevo 3100 Imaging System (FUJIFILM VisualSonics, Toronto, Ontario, Canada) with an MX400 line ultrasound transducer (30 MHz). Firstly, the mice were fixed after 1.5% isopentane anesthesia. Subsequently, the hair of the mice in the chest area was removed. Last, the cardiac ejection fraction (EF) % was measured based on three or more cardiac cycles.

### 2.4. Hematoxylin-Eosin (HE) Staining and Immunohistochemistry

The mouse hearts were fixed overnight with 4% paraformaldehyde after being washed with PBS buffer. Then, the myocardial tissue was embedded in wax and cut into sections. Subsequently, the section was dewaxed and rinsed with alcohol and distilled water. Finally, the section was stained with hematoxylin and eosin, dehydrated, and sealed, which was followed by observing under a microscope.

In order to evaluate the changes of the 4-hydroxynonenal (4-HNE) level in the heart tissue among the different groups, we performed immunohistochemical staining. Firstly, the dewaxed heart sections were blocked with PBS buffer containing 5% goat serum and 1% BSA. Then, the sections were incubated overnight with anti-4-HNE antibody (1 : 200; ab46545; Abcam, Cambridge, UK) at 4°C. On the second day, the section was incubated with an anti-rabbit secondary antibody (1 : 500) for 1 hour. Finally, the sections were observed under a microscope.

### 2.5. Cell Viability

The cell viability was measured using the CCK-8 kit (CK04; Dojindo, Kumamoto, Japan). Firstly, HL-1 cells were plated into 96-well plates and cultured for 24 hours. After the cells were transfected or treated completely, 10 *μ*l CCK-8 was mixed in and maintained at 37°C for 1-2 hours. Finally, the absorbance value of the solution was assessed with a microplate reader (BioTek, Winooski, USA).

### 2.6. Malondialdehyde (MDA) and Glutathione (GSH) Measurement

According to the manufacturer's manuals, MDA levels and GSH levels in tissues or cells were detected with a MDA detection kit (S0131; Beyotime, Shanghai, China) and GSH kit (S0053; Beyotime, Shanghai, China), respectively.

### 2.7. Reactive Oxygen Species (ROS) Measurement

Cellular ROS was evaluated with DCFH-DA (D6883; Sigma-Aldrich, MS, USA), a fluorescent probe [25, 26]. DCFH-DA can enter living cells and transform them into nonfluorescent DCFH, and intracellular reactive oxygen species can convert DCFH into green fluorescent DCF. Therefore, the higher the green fluorescence level, the higher the intracellular ROS level. HL-1 cells were plated into 12-well plates and washed three times with PBS after transfection or treatment. Then, 1 ml DCFH-DA solution (10 *μ*M) was added to each well at 37°C in the dark for 30 min. After three washes, 4% paraformaldehyde was used to fix cells. Finally, the cells were incubated with a DAPI solution to stain the nuclei and then estimated under a fluorescence microscope.

### 2.8. Western Blotting Analysis

To obtain proteins of HL-1 cells or cardiac tissues, a RIPA lysis buffer (89901; Thermo Fisher, IL, USA) containing 1% protease and phosphatase inhibitor (78442; Thermo Fisher, IL, USA) was used to lyse cells and cardiac tissue. Protein concentration was quantified using a BCA assay kit (23225; Thermo Fisher, IL, USA). After quantification, 5× sodium dodecyl sulfate (SDS) protein loading buffer (P0015L; Beyotime, Shanghai, China) was mixed with the protein lysates, and then the protein mix was denatured at 95°C for 10 min. The same amount of protein was loaded to sodium dodecyl sulfate-polyacrylamide gel electrophoresis (SDS-PAGE) gels for electrophoresis and then transferred to a polyvinylidene difluoride (PVDF) membrane (Millipore, CA, USA). The PVDF membrane was blocked with a blocking buffer (P0252; Beyotime, Shanghai, China) at room temperature for 30 min, followed by incubating with a primary antibody (*β*-tubulin and MyD88 (2146 and 4283, Cell Signaling Technology, MA, USA) antibody) at 4°C overnight. The next day, the membrane was detected with a chemiluminescence system (GE AI600, MA, USA) after incubating with the HRP-conjugated secondary antibody.

### 2.9. Quantitative Real-Time Polymerase Chain Reaction (qRT-PCR)

The total RNA of HL-1 cells or myocardial tissue was extracted by TRIzol (15596026; Invitrogen) and reverse transcribed into cDNA using a PrimeScript™ RT reagent kit (RR047; Takara, Kyoto, Japan). According to the manufacturer's manuals, qRT-PCR was performed through the CFX96 Real-Time PCR System (Bio-Rad, CA, USA) using TB Green Premix Ex Taq II (RR820L; Takara, Kyoto, Japan). The 2^-*ΔΔ*t^ method was used for relative quantification of miRNA expression. Primers for miRNA were synthesized with the Servicebio Technology Co., Ltd. (Wuhan, China). qRT-PCR of miR-340-5p used miRNA-specific stem-loop primers, and U6 was set as its internal reference. The primers are as follows for miR-340-5p: 5′-CTCAACTGGTGTCGTGGAGTCGGCAATTCAGTTGAGAATCAGTC-3′ (RT primer), 5′-ACACTCCAGCTGGGTTATAAAGCAATGAGA-3′ (forward primer), and 5′-TGGTGTCGTGGAGTCG-3′ (reverse primer), and U6: 5′-CTCGCTTCGGCAGCACA-3′ (forward primer) and 5′-AACGCTTCACGAATTTGCGT-3′ (reverse primer).

### 2.10. Dual-Luciferase Reporter Assay

TargetScan software (http://www.targetscan.org/) was used to predict the possible binding site of miR-340-5p with MyD88. The 3′UTR region of wild-type MyD88 was inserted into a psiCHECK2 plasmid (Geneseed; Guangzhou, China) to produce the wild-type MyD88 (wt-MyD88) reporter plasmid. Similarly, the 3′UTR region of the mutant MyD88 was inserted into the psiCHECK2 plasmid (Geneseed; Guangzhou, China) to construct the mutant MyD88 (mut-MyD88) reporter plasmid on the basis of the possible binding site. miR-340-5p mimic and mimic-NC were purchased from GenePharma (Shanghai, China). miR-340-5p mimic or mimic-NC was cotransfected with the wt-MyD88 reporter or mut-MyD88 reporter plasmid into HL-1 cells using the Lipofectamine 2000 kit (11668-027; Invitrogen, CA, USA). 48 hours after the HL-1 cell transfection, the luciferase activity was assessed based on a double-luciferase reporter assay kit (FR201; TransGen, Beijing, China).

### 2.11. RNA Pull-Down

In order to further verify the interaction between miR-340-5p and MyD88, the RNA pull-down assay was performed according to the method described in previous studies [27, 28]. Briefly, Bio-miR-340-5p or Bio-NC (GenePharma; Shanghai, China) was transfected into HL-1 cells based on the Lipofectamine 2000 kit. 48 hours after transfection, streptavidin-coated magnetic beads (11205D; Invitrogen, CA, USA) were incubated with the lysates of the above cells to pull down the biotin-bounded RNA complex. MyD88 expression bounded with Bio-miR-340-5p or Bio-NC was quantitatively detected via qRT-PCR analysis and normalized to the “input” control. MyD88: 5′-GTGAGCTCATCGAAAAGAGGTGC-3′ (forward primer) and 5′-TGGAGAGAGGCTGAGTGCAA-3′ (reverse primer).

### 2.12. Statistical Analysis

SPSS 25.0 (SPSS, IL, USA) was applied for data analysis. Figures were drawn using GraphPad Prism 8 software. All the data were expressed as mean value ± SD. The comparison between the two groups used the *t*-test. Comparison among three groups or more used one-way ANOVA analysis. *P* value < 0.05 was determined as statistically significant.

## 3. Results

### 3.1. The Expression Level of miR-340-5p Decreased in the SIC Mouse Model

**Figure 1:**
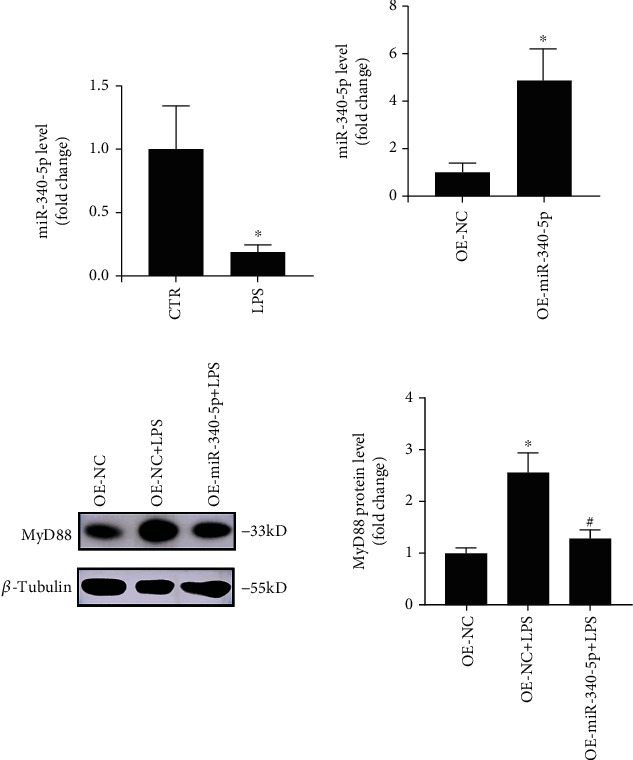
The miR-340-5p level and the effect of miR-340-5p overexpression on the MyD88 level in the SIC mouse model. (a) qRT-PCR assay of the miR-340-5p level in the SIC mouse model (LPS) or control (CTR) group. (b) qRT-PCR assay of the miR-340-5p level in the OE-NC and OE-miR-340-5p group. (c) Western blotting analysis of the MyD88 expression in OE-NC, OE-NC+LPS, and OE-miR-340-5p+LPS group. *N* = 6; ^∗^*P* < 0.05 versus the CTR or OE-NC group; ^#^*P* < 0.05 versus the OE-NC+LPS group.

To observe the miR-340-5p level in the myocardial tissue of the SIC mouse model, we have constructed the SIC model and control group by intraperitoneally injecting with LPS (10 mg/kg) and PBS for 6 hours, respectively. Subsequently, we evaluated the miR-340-5p level using the qRT-PCR method. As shown in Figure 1(a), the miR-340-5p expression level was decreased significantly in the SIC mouse model.

### 3.2. miR-340-5p Overexpression Alleviated the Increase of MyD88 Level in SIC Mouse Model

To investigate whether the overexpression of miR-340-5p affects the expression of MyD88 in vivo, we pretreated with AAV-9 pre-miR-340-5p or AAV-9 negative control via intramyocardial injection for 21 days in the mouse and constructed a SIC model with LPS treatment for 6 hours at day 21. Firstly, compared with the OE-NC group, we verified that the miR-340-5p level was increased in the OE-miR-340-5p group (Figure 1(b)). Then, we have observed that the MyD88 expression was enhanced via LPS treatment, and overexpression of miR-340-5p partly reduced this tendency (Figure 1(c)).

### 3.3. miR-340-5p Overexpression Alleviated the Oxidative Stress Injury in the SIC Mouse Model

**Figure 2:**
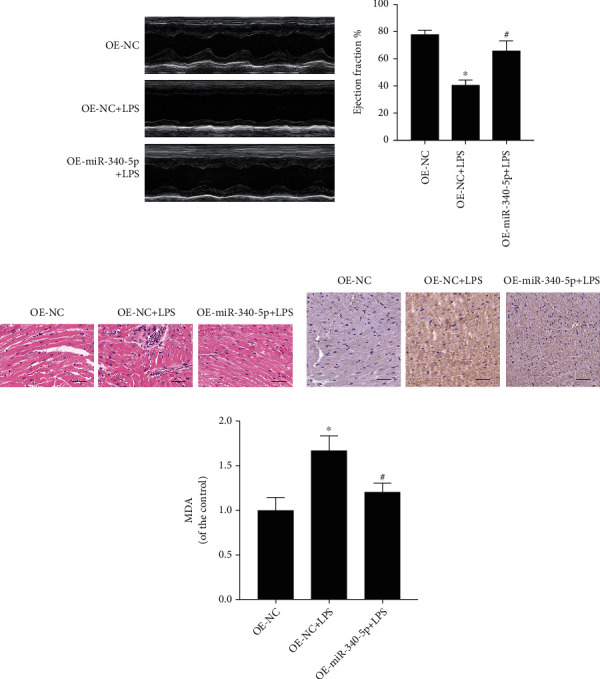
The effect of the miR-340-5p overexpression on cardiac function, pathology, and oxidative stress level in the SIC mouse model. (a–d) The echocardiography analysis (a), hematoxylin-eosin (HE) staining (b), 4-HNE immunohistochemistry staining (c), and MDA assay (d) in OE-NC, OE-NC+LPS, or OE-miR-340-5p+LPS group. *N* = 6; scale bar: 100 *μ*m; ^∗^*P* < 0.05 versus the OE-NC group; ^#^*P* < 0.05 versus the OE-NC+LPS group.

To illuminate whether the overexpression of miR-340-5p affects oxidative stress injury in vivo, we performed echocardiography and HE staining, detected the MDA and 4-HNE levels in the heart. The EF% was reduced in the SIC model, and the overexpression of miR-340-5p relieved this change (Figure 2(a)). Besides, HE staining showed that the miR-340-5p overexpression could relieve the myocardial damage (cell swelling and inflammatory cell infiltration) in the SIC mouse model (Figure 2(b)). Moreover, the miR-340-5p overexpression improved the high oxidative stress level (4-HNE and MDA) in the SIC model (Figures 2(c) and 2(d)).

### 3.4. The miR-340-5p Level Was Reduced in the SIC Cell Model

**Figure 3:**
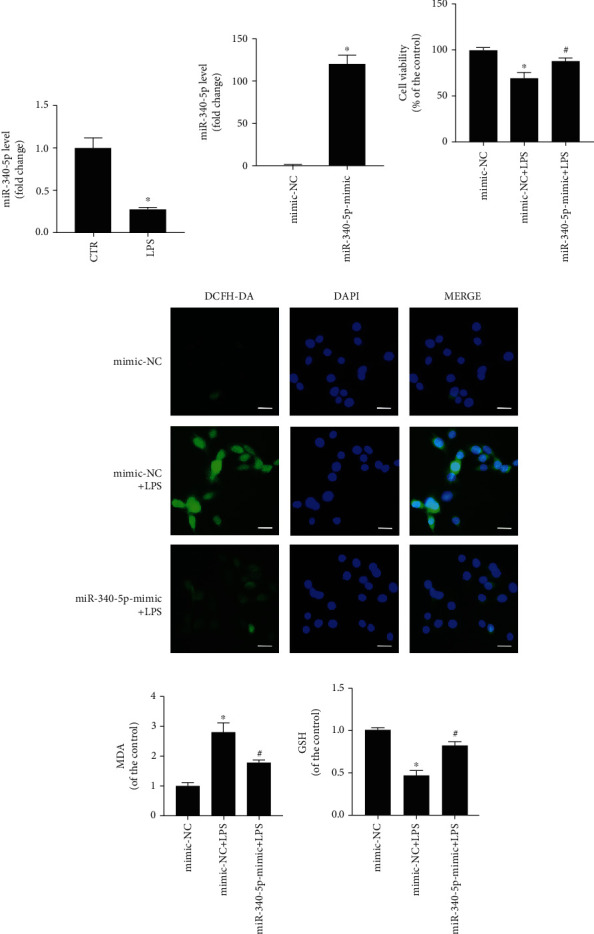
The miR-340-5p level and the effect of the miR-340-5p overexpression on oxidative stress injury in the SIC cell model. (a) qRT-PCR assay of the miR-340-5p level in the SIC cell model (LPS) or control (CTR) group. (b) qRT-PCR assay of the miR-340-5p level in the mimic-NC or miR-340-5p-mimic group. (c–f) The cell viability (c), ROS level (d), MDA assay (e), and GSH assay (f) in the mimic-NC, mimic-NC+LPS, or miR-340-5p-mimic+LPS group. *N* = 3; scale bar: 20 *μ*m; ^∗^*P* < 0.05 versus the CTR or mimic-NC group; ^#^*P* < 0.05 versus the mimic-NC+LPS group.

To evaluate the miR-340-5p level in the SIC cell model, we have constructed the SIC cell model and control group by administering with LPS (10 *μ*g/ml) and PBS treatment for 6 hours, respectively. Consistent with the results of the SIC animal model, it was found that the miR-340-5p expression level also decreased in the SIC cell model (Figure 3(a)).

### 3.5. miR-340-5p Mimic Pretreatment Improved the Oxidative Stress Injury in the SIC Cell Model

To explore whether miR-340-5p has a role in oxidative stress injury in the SIC cell model, we constructed miR-340-5p overexpression cells or control via using the miR-340-5p mimic or mimic-NC pretreatment, followed by building a SIC cell model with LPS (10 *μ*g/ml) treatment for 6 hours. The four groups of cells were listed as follows: control group (mimic-NC group), LPS group (mimic-NC+LPS group), miR-340-5p mimic group (miR-340-5p-mimic group), and miR-340-5p mimic-combined LPS group (miR-340-5p-mimic+LPS group). In the mimic-NC group, the HL-1 cell was pretreated with mimic-NC for 48 hours, then administered with PBS for 6 hours. The cells in the mimic-NC+LPS group were pretreated with mimic-NC for 48 hours, followed by administration with LPS (10 *μ*g/ml) for 6 hours. In the miR-340-5p-mimic group, the HL-1 cell was pretreated with miR-340-5p mimic for 48 hours, followed by administration with PBS for 6 hours. The cells in the miR-340-5p-mimic+LPS group were pretreated with miR-340-5p mimic for 48 hours, followed by administration with LPS (10 *μ*g/ml) for 6 hours. Compared with the mimic-NC group, the expression level of miR-340-5p was enhanced significantly in the miR-340-5p-mimic group (Figure 3(b)). Then, we demonstrated that the cell viability was decreased after LPS treatment, and the miR-340-5p improved this change (Figure 3(c)). As we all know, ROS is the main culprit of oxidative stress. In this research, we used DCFH-DA to detect the level of ROS, and we demonstrated that miR-340-5p mimic pretreatment could decrease the ROS level in the SIC cell model (Figure 3(d)). Similarly, the MDA, a type of oxidative stress product, was enhanced in the SIC cell model, and the miR-340-5p mimic pretreatment could partly relieve it (Figure 3(e)). Conversely, the GSH, a type of antioxidant, was downregulated in SIC, and the miR-340-5p mimic pretreatment could partly relieve it (Figure 3(f)).

### 3.6. miR-340-5p Inhibitor Pretreatment Further Promoted the Oxidative Stress Injury in the SIC Cell Model

**Figure 4:**
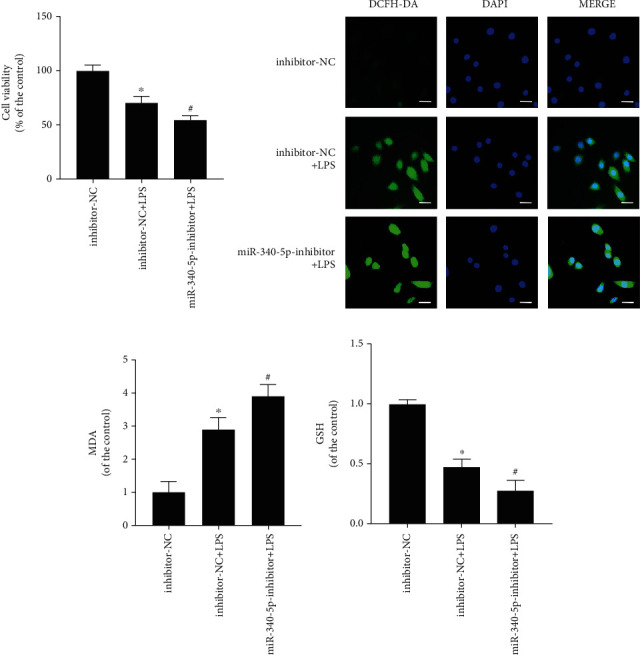
The effect of miR-340-5p knockdown on oxidative stress injury in the SIC cell model. (a–d) The cell viability (a), ROS level (b), MDA assay (c), and GSH assay (d) in the inhibitor-NC, inhibitor-NC+LPS, or miR-340-5p-inhibitor+LPS group. *N* = 3; scale bar: 20 *μ*m; ^∗^*P* < 0.05 versus the inhibitor-NC group; ^#^*P* < 0.05 versus the inhibitor-NC+LPS group.

To further verify the influence of miR-340-5p on oxidative stress injury in the SIC cell model, we constructed the miR-340-5p inhibitor-treated cells and control group with the miR-340-5p inhibitor and inhibitor-NC, respectively. And then, we built a SIC cell model with LPS treatment. The three groups of cells were listed as follows: control group (inhibitor-NC group), LPS group (inhibitor-NC+LPS group), and miR-340-5p inhibitor-combined LPS group (miR-340-5p-inhibitor+LPS group). In the inhibitor-NC group, the HL-1 cell was treated with inhibitor-NC for 48 hours, followed by administration with PBS for 6 hours. The inhibitor-NC+LPS group cells were pretreated with inhibitor-NC for 48 hours, followed by administration with LPS (10 *μ*g/ml) for 6 hours. The cells in the miR-340-5p-inhibitor+LPS group were pretreated with miR-340-5p inhibitor for 48 hours and then administered with LPS (10 *μ*g/ml) for 6 hours. Apparently, the treatment of the miR-340-5p inhibitor further promoted cell viability injury mediated by LPS (Figure 4(a)). Besides, the ROS and MDA levels were further increased in the SIC cell model through the miR-340-5p inhibitor pretreatment (Figures 4(b) and 4(c)). Furthermore, the GSH level was further decreased via miR-340-5p inhibitor pretreatment (Figure 4(d)).

### 3.7. miR-340-5p Negatively Regulated the MyD88 Level

**Figure 5:**
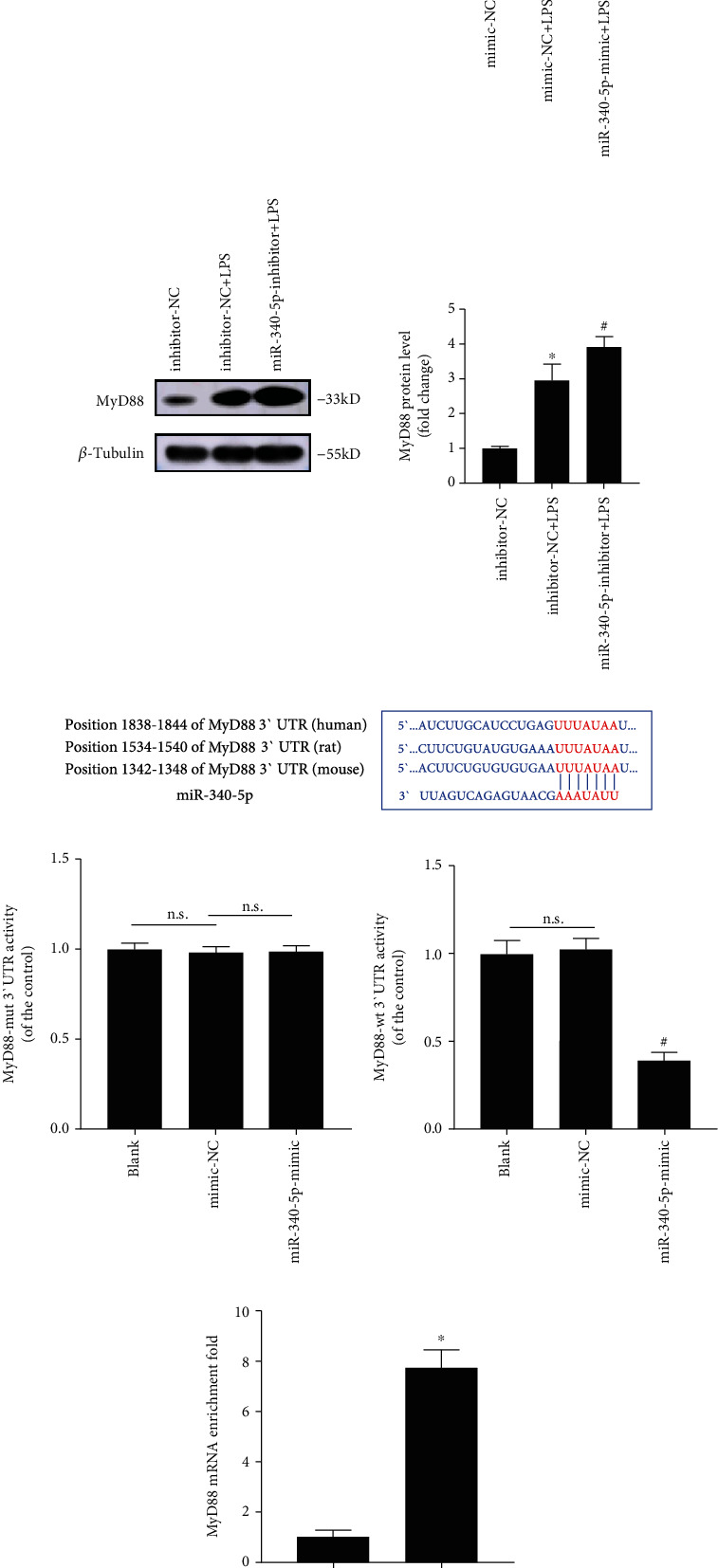
The effect of miR-340-5p overexpression or knockdown on the MyD88 level and the interaction of miR-340-5p with MyD88 mRNA. (a) Western blotting analysis of MyD88 expression in the mimic-NC, mimic-NC+LPS, or miR-340-5p-mimic+LPS group. (b) Western blotting analysis of the MyD88 level inhibitor-NC, inhibitor-NC+LPS, or miR-340-5p-inhibitor+LPS group. (c) The predicted binding sites of miR-340-5p with the 3′UTR of MyD88 in different species. (d, e) The dual-luciferase reporter activities of MyD88-mut 3′UTR (d) or MyD88-wt 3′UTR (e) in blank, mimic-NC, or miR-340-5p group. (f) Detection of MyD88 enrichment on Bio-NC or Bio-miR-340-5p in HL-1 cells via RNA pull-down assay. *N* = 3; ^∗^*P* < 0.05 versus mimic-NC (a), inhibitor-NC (b), or Bio-NC group (f); ^#^*P* < 0.05 versus mimic-NC+LPS (a), inhibitor-NC+LPS (b), or mimic-NC group (e). n.s.: no significant; mut: mutant; wt: wild type.

To explore whether miR-340-5p affects the MyD88 level in vitro, we used Western blotting analysis to observe the change of MyD88 after the miR-340-5p mimic or inhibitor pretreatment in the SIC model. We have observed that miR-340-5p mimic and inhibitor pretreatment could reduce and further increase the MyD88 level in the SIC cell model, respectively (Figures 5(a) and 5(b)). Using the TargetScan software, we predicted that the 3′UTR of MyD88 has the same bind site with miR-340-5p in different species (Figure 5(c)). Dual-luciferase reporter assay confirmed the interaction between miR-340-5p and MyD88 (Figures 5(d) and 5(e)). Moreover, the RNA pull-down assay showed that the biotinylated miR-340-5p could pull down endogenous MyD88 mRNA in HL-1 cells (Figure 5(f)). Therefore, the miR-340-5p can directly and negatively regulate the expression of MyD88.

### 3.8. MyD88 Knockdown Could Decrease the Oxidative Stress Injury in the SIC Cell Model

**Figure 6:**
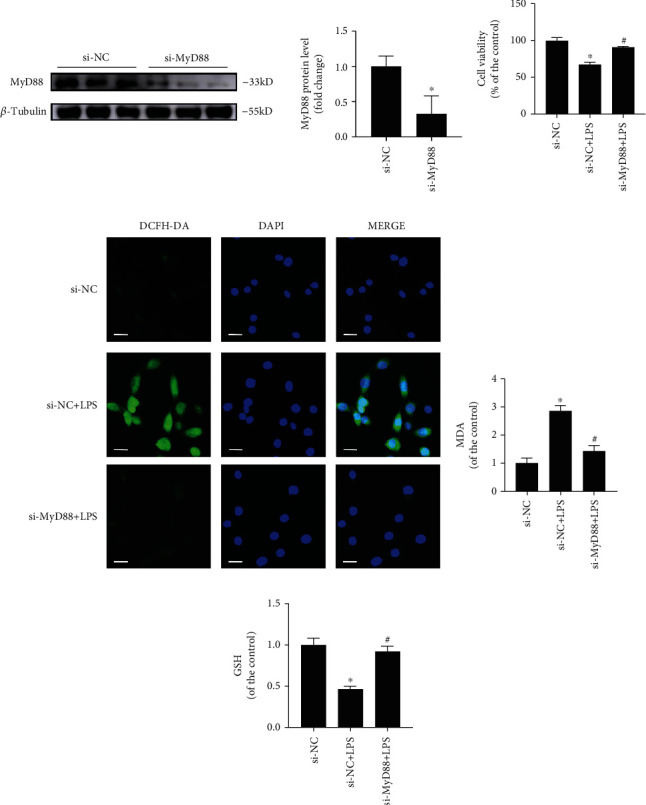
The effect of MyD88 siRNA on oxidative stress injury in the SIC cell model. (a) Western blotting analysis of MyD88 expression in the si-NC and si-MyD88 groups. (b–e) The cell viability (b), ROS level (c), MDA assay (d), and GSH assay (e) in si-NC, si-NC+LPS, or si-MyD88+LPS group. *N* = 3; scale bar: 20 *μ*m; ^∗^*P* < 0.05 versus the si-NC group; ^#^*P* < 0.05 versus the si-NC+LPS group.

To further investigate the role of MyD88 in the oxidative stress injury of the SIC cell model, we constructed the MyD88 knockdown cells or control with the MyD88 si-RNA or si-NC pretreatment and then built a SIC cell model with LPS treatment. The cells were divided into four groups: the control group (si-NC group), LPS group (si-NC+LPS group), MyD88 siRNA group (si-MyD88 group), and MyD88 siRNA-combined LPS group (si-MyD88+LPS group). Cells in the si-NC group were pretreated with si-NC for 48 hours and then administered with PBS for 6 hours. The si-NC+LPS group cells were pretreated with si-NC for 48 hours, followed by administration with LPS (10 *μ*g/ml) for 6 hours. In the si-MyD88 group, HL-1 cells were pretreated with si-MyD88 for 48 hours and then administered with PBS for 6 hours. The si-MyD88+LPS group cells were pretreated with si-MyD88 for 48 hours and then administered with LPS (10 *μ*g/ml) for 6 hours. First, we observed that the MyD88 siRNA treatment could reduce the MyD88 level (Figure 6(a)). Then, we have shown that the MyD88 siRNA treatment alleviated the cell viability injury induced by LPS (Figure 6(b)). In addition, we have found that the ROS and MDA levels were decreased in the SIC cell model after MyD88 siRNA treatment (Figures 6(c) and 6(d)). Furthermore, the GSH level was upregulated after MyD88 siRNA treatment (Figure 6(e)).

## 4. Discussion

SIC is related to the rising mortality in septic shock patients, but there is still no specific drug for its treatment [5]. Previous studies demonstrated that the mechanisms of SIC are varied, which may be related to inflammatory response, oxidative stress injury, mitochondrial injury, and cardiomyocyte death [29, 30]. Our research sought to clarify the role and mechanisms of miR-340-5p in SIC to explore the new target of treatment. In this research, we built a SIC animal model using LPS-treated mice and observed that the miR-340-5p level was reduced and the MyD88 level was increased. Besides, further study confirmed that miR-340-5p overexpression protects SIC from oxidative stress injury. Subsequently, we used LPS to treat the HL-1 cell line to construct the SIC cell model, and we found that the miR-340-5p mimic and inhibitor can alleviate and promote the oxidative stress injury induced by LPS, respectively. Moreover, we also observed that miR-340-5p mimic treatment could inhibit the upregulation of MyD88 expression induced by LPS, while the miR-340-5p inhibitor had the opposite effect. Dual-luciferase reporter assay and RNA pull-down assay further confirmed that miR-340-5p functions by directly binding to MyD88. Finally, we demonstrated that pretreatment with si-MyD88 in the SIC cell model can also reduce oxidative stress injury. Therefore, the results above indicated that miR-340-5p alleviates SIC by targeting MyD88-mediated oxidative stress injury.

miR-340-5p was involved in various pathophysiological processes such as inflammation, tumorigenesis, and oxidative stress [16, 31, 32]. Besides, it has also shown that miR-340-5p plays diverse roles in different states of cardiovascular disease [14–18]. In chronic heart failure, the miR-340-5p level is upregulated, which can promote the progress of heart failure [17, 18]. On the contrary, the expression of miR-340-5p in acute myocardial infarction or ischemia-reperfusion models was downregulated, and it could improve myocardial injury via regulating Act1, NF-κB, or HDAC [14–16]. In this study, we demonstrated that the miR-340-5p level was reduced in the LPS-induced SIC model. Moreover, the results of further overexpression and knockdown experiments suggest that miR-340-5p protected the SIC from oxidative stress injury. In all, our results confirmed that miR-340-5p has a protective role in the SIC, which may provide new opinions for the diagnosis and treatment of SIC in the future.

The MyD88 molecule combines with serine/threonine kinase and interleukin-1 receptor-related kinases 2 and 4 to form Myddosome, which is of vital importance in Toll-like receptor-mediated innate and acquired immune responses [20, 21, 33]. A previous study has found that high circulating MyD88 levels are related to the poor prognosis in patients with neonatal sepsis [22]. Besides, in other sepsis-induced organ (lung, kidney, and liver) injury models, inhibition of MyD88 can improve the damage by reducing inflammation [34–36]. In addition, the knockout of MyD88 can significantly improve cardiac dysfunction, systemic inflammation, and mortality in the SIC mouse model [37]. Similarly, another study showed that blocking MyD88 by injecting MyD88-neutralizing antibodies into the blood circulation of SIC mice can reduce the release of cytokines, neutrophil infiltration, and apoptosis of the myocardium, so as to play a protective role on the myocardium [38]. Similarly, we also observed that the MyD88 level was increased in SIC, and knockdown of MyD88 could improve oxidative stress injury in SIC cardiomyocytes.

SIC cardiomyocytes produce a large amount of ROS, which can increase the level of oxidative stress and reduce cell viability [39, 40]. Huang et al. found that the production of oxidative stress markers (MDA, ROS, and lactate dehydrogenase (LDH)) increased in LPS-administered cardiomyocytes, and knocking down UCP2 can further aggravate the level of oxidative stress injury [41]. Besides, Joseph et al. showed that LPS-treated adult mouse cardiomyocytes in vitro could increase cellular ROS and mitochondrial superoxide, while the cardiac function and the level of oxidative stress of isolated cardiomyocytes in NOX2 knockdown mice administered by LPS had no significant change, suggesting that NOX2 knockout could improve the cardiac function by alleviating the oxidative stress injury [42]. Similarly, our research also indicated that the level of oxidative stress increased and the cardiac function decreased in the SIC mouse model, while the miR-340-5p overexpression plays a protective role. In in vitro experiments, miR-340-5p mimic pretreatment can alleviate the oxidative stress injury induced by LPS, suggesting that miR-340-5p can protect myocardial function by reducing the level of myocardial oxidative stress. In our previous bioinformatics analysis, we demonstrated that MyD88 may be the hub gene of SIC [23], and the predictive analytics showed that MyD88 is the target gene of miR-340-5p. In order to further investigate whether miR-340-5p affects the level of myocardial oxidative stress by regulating MyD88 in SIC, we confirmed the interaction of miR-340-5p with MyD88 via the dual-luciferase reporter assay and RNA pull-down assay. Moreover, we have also observed that the miR-340-5p mimic and inhibitor could decrease and further enhance the MyD88 expression in the SIC cell model, respectively. In addition, we found that the MyD88 knockdown could also relieve the oxidative stress injury mediated by LPS. In sum, the results above suggest that miR-340-5p can alleviate oxidative stress injury by targeting MyD88 in SIC.

It should be pointed out that there are still some limitations in the current study. First, the level of miR-340-5p in clinic samples of SIC patients was not verified. We will evaluate the miR-340-5p level of the SIC blood sample and its clinical significance in the future study. Second, we have not studied the downstream pathway of the MyD88 in our study. Many studies have demonstrated that the MyD88/NF-*κ*B signaling pathway plays an important role in the oxidative stress injury process [43–45]. Besides, Zhou et al. have found that the inhibition of MyD88/MAPK/mTOR pathway can alleviate the LPS mediated-intestinal oxidative stress injury [46]. The above studies indicated that the NF-*κ*B and MAPK may be involved in the downstream pathway of MyD88-mediated oxidative stress injury. Therefore, we will carry out more in vivo and in vitro experiments to clarify the detailed molecular mechanism of SIC in our future research.

## 5. Conclusion

In this research, we studied the role of miR-340-5p in SIC to obtain a new target of the treatment. Our study demonstrated that miR-340-5p can improve myocardial oxidative stress injury by targeting MyD88 in SIC, which may contribute to a new treatment opinion in the future.

## Figures and Tables

**Table 1:** List of miR-340-5p mimic and inhibitor and MyD88 siRNA sequences.

Name	Species	Sequence
miR-340-5p mimic	Mouse	Sense 5′-UUAUAAAGCAAUGAGACUGAUU-3′ Antisense 5′-UCAGUCUCAUUGCUUUAUAAUU-3′
mimic-NC	Mouse	Sense 5′-UUCUCCGAACGUGUCACGUTT-3′ Antisense 5′-ACGUGACACGUUCGGAGAATT-3′
miR-340-5p inhibitor	Mouse	5′-AAUCAGUCUCAUUGCUUUAUAA-3′
inhibitor-NC	Mouse	5′-CAGUACUUUUGUGUAGUACAA-3′
MyD88 siRNA	Mouse	Sense 5′-CUGCGGUUCAUCACUAUAUTT-3′ Antisense 5′-AUAUAGUGAUGAACCGCAGTT-3′
si-NC	Mouse	Sense 5′-UUCUCCGAACGUGUCACGUTT-3′ Antisense 5′-ACGUGACACGUUCGGAGAATT-3′

## Data Availability

The original data can be obtained from the corresponding author if it is permitted by all authors.
